# Comparison of Real Time IS*6110-*PCR, Microscopy, and Culture for Diagnosis of Tuberculous Meningitis in a Cohort of Adult Patients in Indonesia

**DOI:** 10.1371/journal.pone.0052001

**Published:** 2012-12-21

**Authors:** Lidya Chaidir, Ahmad Rizal Ganiem, Adri vander Zanden, Soni Muhsinin, Tina Kusumaningrum, Inri Kusumadewi, Andre van der Ven, Bachti Alisjahbana, Ida Parwati, Reinout van Crevel

**Affiliations:** 1 Health Research Unit, Faculty of Medicine, Padjadjaran University/Hasan Sadikin Hospital, Bandung, Indonesia; 2 Department of Neurology, Faculty of Medicine, Padjadjaran University/Hasan Sadikin Hospital, Bandung, Indonesia; 3 Laboratory for Medical Microbiology and Public Health, Enschede, The Netherlands; 4 Department of Clinical Pathology, Faculty of Medicine, Padjadjaran University/Hasan Sadikin Hospital, Bandung, Indonesia; 5 Department of Medicine, Radboud University Medical Centre, Nijmegen, The Netherlands; Institut de Pharmacologie et de Biologie Structurale, France

## Abstract

**Background:**

Bacteriological confirmation of tuberculous (TB) meningitis is difficult. Culture is slow and microscopy has insufficient sensitivity. We evaluated real time PCR targeting insertion sequence IS*6110* among 230 consecutive adult patients with subacute meningitis in a referral hospital in Indonesia.

**Methods:**

Cerebrospinal fluid (CSF) samples were examined using microscopy, solid and liquid culture, and real time IS*6110*-PCR with a fluorescence-labeled probe using DNA extracted from CSF. CSF samples from 40 non-infectious neurology patients were used as negative controls. IS*6110*-PCR results were linked with clinical and CSF characteristics.

**Results:**

Most patients presented with subacute meningitis, after a median of 14 days of symptoms (range 7–30). After exclusion of cryptococcal and bacterial meningitis, 207 patients were classified as definite or probable TB meningitis; 17.9% with HIV infection. Among this group IS*6110*-PCR gave the highest positivity rate (68%, 95% CI 62–74%) compared with microscopy of ZN-stained slides (11%, 95% CI 7–15%), and mycobacterial culture using solid (36%, 95% CI 29–42%) and liquid (44%, 95% CI 37–51%) media. IS*6110*-PCR was positive in 92% of patients with culture-positive and 42% of patients with culture-negative probable TB meningitis. Among culture-negative patients, a positive PCR was associated with a history of TB treatment, a longer duration of illness, a higher CSF cell count and protein, and a lower CSF glucose. IS*6110*-PCR was negative in all CSF samples from non-meningitis control patients.

**Conclusions:**

Real time IS*6110*-PCR is a quick, sensitive, and specific test for diagnosing of TB meningitis in this setting. Its performance in other (less-developed) settings needs further study.

## Introduction

Tuberculous (TB) meningitis is the most severe form of tuberculosis and causes substantial morbidity and mortality in adults and children [1]. Early recognition and treatment of the disease is believed to be able to reduce the burden of this disease, but this is hampered by the fact that it is often difficult to find bacteriological proof for TB meningitis [2], [3]. Microscopy of cerebrospinal fluid (CSF), although inexpensive and rapid, has a poor sensitivity, ranging from 1.9% to 20% in different series, with the exception of one study (58%) that used large volumes of CSF [3], [4], [5]. CSF culture, also lacks sensitivity for diagnosing TB meningitis [2], [3], [4], [6]. Furthermore, the slow growth of *Mycobacterium tuberculosis,* that usually takes up to 4 to 6 weeks limits the role of culture in decisions regarding initiation of TB treatment [7]. Therefore, a rapid and accurate diagnostic test would greatly benefit timely and adequate management of patients with possible TB meningitis.

Nucleic acid amplification (NAA) tests seem an attractive diagnostic tool for TB meningitis because of their speed and expected high sensitivity. However, a systematic review and metaanalysis of the accuracy of NAA tests for diagnosis of TB meningitis showed that commercial NAA test had a high specificity (98%, 95% CI 97–99%) but a low sensitivity when compared with culture (56%, 95% CI 46–66%) among 14 studies combined [8]. The recently developed GeneXpert system, combining DNA extraction with a real time PCR that simultaneously detects both *M. tuberculosis* and rifampin resistance, has a lower sensitivity compared to culture, but to our knowledge only one study has reported use in suspected meningitis [9]. In that study none of 19 CSF samples were positive with GeneXpert. This method was not available in Indonesia when the study was performed.

In-house PCR for diagnosis of TB meningitis may be more sensitive, possibly due to the use of nested PCR, DNA extraction methods, or use of different molecular targets. However, the precise role of in-house PCR for TB meningitis remains uncertain. Many in-house assays have been evaluated without adequate standardization and using small groups of patients [10], [11], [12], [13]. The present study therefore evaluated in-house real time PCR targeting IS*6110* in CSF samples from a well-characterized cohort of 230 adult patients with suspected meningitis, making comparisons with CSF *M. tuberculosis* culture and microscopy.

## Methods

### Ethics Statement

Anonymized CSF samples were used from an already-existing hospital collection, collected as part of a project ‘Optimization of diagnosis of meningitis’, approved by the Ethical Committee of Hasan Sadikin Hospital/Faculty of Medicine of Universitas Padjadjaran, Bandung, Indonesia (No. 85/FKUP-RSHS/KEPK/Kep/EC/2006). The current study made use of an already existing sample collection, no separate patient consent was asked for this study. HIV testing is done routinely after verbal informed consent for all patients with suspected meningitis in Hasan Sadikin hospital. Consent is obtained from closest relatives (husband/wife or parents) for those patients who are unstable or unconscious at time of presentation. HIV testing was done anonymously afterwards for those who had died before consent could be obtained. This study was approved by the ethical review board of Padjadjaran University/Hasan Sadikin Hospital, Bandung, Indonesia.

### Setting and Patients

Patients were recruited at Hasan Sadikin Hospital, top referral hospital for West Java, Indonesia, where approximately 100 adult patients present with subacute meningitis each year. A clinical diagnosis of meningitis in this setting is based on clinical findings, CSF criteria or both. Clinical criteria of meningitis included headache, fever and neck stiffness, with or without altered consciousness. CSF criteria were cell count >10 cell/mm3, protein concentration >45 mg/dL, or the CSF:blood glucose ratio <0.5; either alone or in combination. For this study, definite TB meningitis was defined as CSF microscopy or culture positive for *M. tuberculosis*. Probable TB meningitis was defined as meningitis with typical CSF findings in conjunction with either: suggestive chest X-ray abnormalities; suggestive TB lymphadenitis; bacteriologically confirmed TB outside the CNS. Definite bacterial meningitis was diagnosed if bacteria were detected in the CSF, while probable bacterial meningitis was diagnosed in patients with characteristic CSF findings without a confirmatory bacteriological result. Cryptococcal meningitis was diagnosed if either India Ink examination or cryptococcal antigen testing of CSF were positive [14]. Final diagnoses was reviewed by a panel of experts. Patients with suspected or proven bacterial meningitis are prescribed ceftriaxone with corticosteroids, those with cryptococcal meningitis amphotericin B, and those with presumed TB standard TB treatment with adjunctive corticosteroids according to national and international guidelines [15].

### Clinical Evaluation

All consecutive patients presenting with clinical meningitis between 2006 and 2008 were included for this study. Signs and symptoms were recorded using standardized forms, and CSF was obtained for routine examination and microbiological testing as described below. Further routine examinations included chest X-ray examination and sputum examination if indicated. Neuroradiology is not routinely done in this setting since it is not covered by the government health insurance for the poor. For this study the HIV-status was determined anonymously for those patients who had died before consent was obtained.

### Routine CSF Examination

Following a lumbar puncture, 5–10 ml CSF was obtained, transported to the laboratory within one hour, and divided into two tubes: one (0.5–1 mL) for CSF cells, protein and glucose, and one (8–10 ml) for microbiological testing. The second tube was then concentrated by centrifugation at 3000×g for 10 minutes. The CSF sediment was used to prepare smears for direct examination of acid-fast bacilli after Ziehl-Neelsen (ZN) staining, bacteria (Gram) and Cryptococci (India ink). For Mycobacterial culture, the sediment was inoculated onto two slants of Ogawa egg medium and on MB/BacT alert system (Biomerieux, Durham, North Carolina, USA) according to the manufacturer’s instruction. Ogawa slants were incubated at 37°C and observed twice weekly for 3 months. For culture of bacterial pathogens some sediment was inoculated on blood agar and Trypton Soy Broth (TSB), while Sabouraud plates were inoculated for growth of fungi. Distilled water was processed in parallel with each CSF sample as a negative control. Cryptococcal antigen (CALAS; Meridian Diagnostics, Cincinnati, Ohio, USA) testing was also done on all CSF samples. At the time of our study, the GeneXpert system was not yet operational in Indonesia.

### Real Time PCR for *M. tuberculosis*

DNA was extracted from 200 µl of CSF sediment by using QIAmp DNA mini kit (Qiagen, USA). CSF samples were spiked with a known concentration of PhHV as an inhibition control. Each batch of extraction included CSF negative controls to rule out any contamination, with a minimum of two negative controls per-18 CSF samples. Primers and probes used for real time IS*6110*-PCR amplify fragment of IS*6110*, a repeated insertion sequence specific for *M. tuberculosis complex* [16]. The total PCR volume was 20 µl (7 µl extracted DNA in 13 µl PCR-mix). The PCR-mix consisted of Platinum Quantitative PCR Supermix-UDG (Invitrogen), 300 nM of each primer of IS*6110*, 150 nM of the FAM-labeled *M. tuberculosis* probe, 200 nM of each primer of PhHV inhibition control, and 150 nM of the Cy5-labeled PhHV inhibition control probe. PCR amplification was carried out during 2 min at 50°C for UDG activation, 10 min at 95°C and 40×15 s at 90°C, 1 min 60°C. Each run of the assay included two negative controls to rule out any contamination along the preparation of PCR-mix and sample addition, respectively. The positive control included the DNA of H37Rv with the Ct value 29±1. PCR was performed in Chromo4^TM^system real time PCR detector (Biorad laboratories, Inc.).

Adequate care was taken to prevent carry over amplicon contamination by performing the PCR in three separate rooms (clean room, extraction room, and amplification room), by using barrier tips, and also by using PCR-mix that contained Uracyl DNA-Glycosylase (UDG). CSF samples from additional 40 non-infectious neurology patients were used as negative controls. For all samples, PCR was done blinded to results of CSF microscopy and culture and clinical data.

### Data Analysis and Statistics

After exclusion of patients with bacterial and cryptococcal meningitis, the positivity rate of microscopy for acid-fast bacilli, *M. tuberculosis* culture using solid and liquid media, and IS*6110*-PCR was expressed as percentage (%) for the remaining group. The sensitivity of IS*6110*-PCR using culture as gold standard was expressed as percentage (95% confidence interval). Comparisons were made of CSF and clinical characteristics of patients after stratification by *M. tuberculosis* culture- and PCR-results. Continuous variables are expressed as mean (SD) if normally distributed and median (interquartile range, IQR) if not normally distributed, and categorical variables as percentage. Differences between groups were compared using Chi-square test for proportions and Mann-Whitney U test for continuous variables, with p-values<0.05 considered statistically significant. Statistical analysis was done by SPSS version 16.

## Results

A total of 230 consecutive patients with suspected meningitis were included. Patients were mostly male (60%), with a median age of 30 years (range 24–36 years), presenting after a median of 14 days (range 7–30 days) of symptoms, mostly with headache (67%) and altered consciousness (31.3%) as chief complaints. On examination, nuchal rigidity (77.2%), lowered consciousness (52.6%), and focal neurological signs were common. HIV infection was present in 22.2%; one patient was not tested.

**Figure 1:**
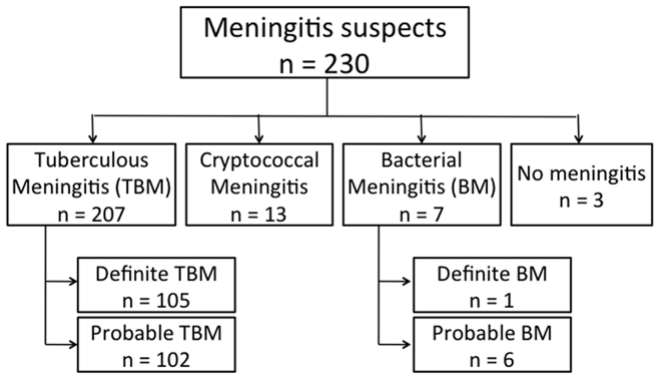
Diagnosis among 230 meningitis suspects based on clinical characteristics and CSF microscopy and culture.

Based on CSF culture and microscopy, subjects were classified into four groups (**figure 1**). TB meningitis was diagnosed in 207 patients (90%), cryptococcal meningitis in 13 (5.6%), bacterial meningitis in 7 (3%), and meningitis was excluded in 3 patients (0.1%). Among 207 patients with suspected TB meningitis, a definite diagnosis could be established in 105 patients (50.7%) based on culture and microscopy (n = 102) and microscopy alone (n = 3). We could classify 102 patients (49.3%) as probable TB meningitis. 17.9% of patients with TB meningitis were HIV-infected.

IS*6110*-PCR assay was positive in 143 patients, 140 patients with TB meningitis, two patients with confirmed cryptococcal meningitis, and one patient with suspected bacterial meningitis. For the patient with suspected bacterial meningitis, TB was also confirmed by direct spoligotyping of the CSF sample for *M. tuberculosis,* as described elsewhere [17]. None of the 40 patients in the control group had a positive IS*6110*-PCR result.

**Figure 2:**
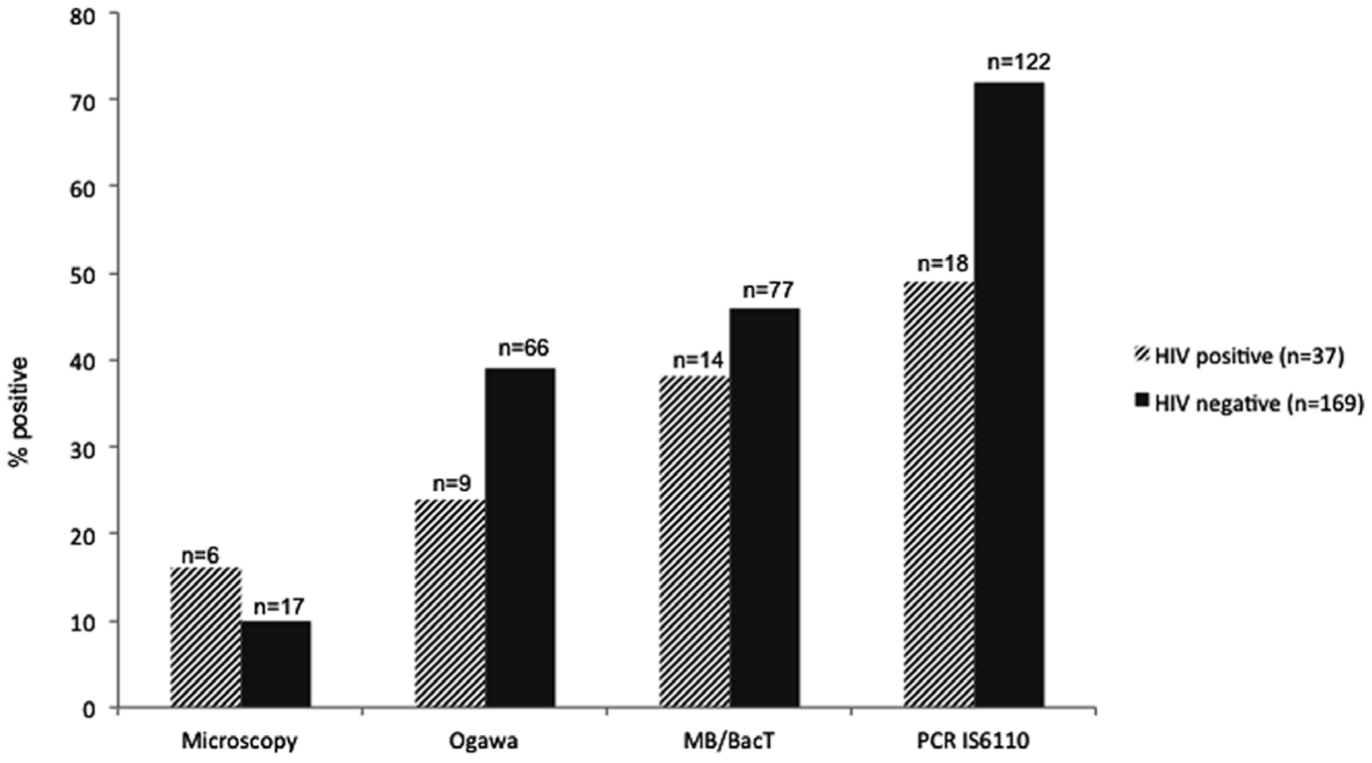
Positivity rate of different diagnostic tests for TB among 207 meningitis patients. Patients with cryptococcal (n = 13), bacterial (n = 7), and no meningitis (n = 3) were excluded, HIV-status was unknown for one patient. ZN staining was used for microscopy, Ogawa for solid culture, and MB/BacT system for liquid culture. Differences between IS*6110*-PCR and solid culture (p<0.001) and microscopy (p<0.001) were statistically significant.

**Figure 2** shows the comparison of diagnostic modalities for TB meningitis. The diagnostic yield of IS*6110*-PCR was much higher (68%, 95% CI 62–74%) compared to solid culture (36%, 95% CI 29–42%; p<0.001) and microscopy (11%, 95% CI 7–15%; p<0.001), and higher than liquid culture (44%, 95% CI 37–51%) although this was not statistically significant (p = 0.262). As shown in **figure 2**, this was true for HIV-infected and -non-infected patients although the positivity yield of IS*6110*-PCR was lower among HIV-infected patients (49%, 95% CI 33–65% vs 73%, 95% CI 66–80%; p = 0.004). Larger CSF samples were more often PCR-positive; 50% of samples <4 mL (n = 22) were positive, compared with 71% of samples >4 mL (n = 181, p = 0.05).

*M. tuberculosis* culture (either solid or liquid) was positive in 102 patients, and IS*6110*-PCR in 140 patients (**table 1**). Using culture as the gold standard, IS*6110*-PCR had a sensitivity of 92% (95% CI 87–97%), compared with 21% (95% CI 13–29%) for microscopy. Among 46 culture-negative PCR-positive patients, 43 were clinically diagnosed as probable TB meningitis. For all of those patients there was supporting evidence for a diagnosis of TB: 17 patients had confirmed TB outside the CNS; 13 were cured after TB treatment; and 13 had been taking TB drugs for some days before lumbar puncture, while it is known that CSF cultures quickly become negative after start of TB treatment.

**Table 1:** Comparison of *M. tuberculosis* culture and IS*6110*-PCR results among 207 TB meningitis patients.

		Culture		Total
		Positive	Negative	
IS*6110*-PCR	Positive	94 (45%)	46 (22%)	140 (67%)
	Negative	8 (4%)	59 (29%)	67 (33%)
	Total	102 (49%)	105 (51%)	207

Among patients with a negative *M. tuberculosis* culture, positive IS*6110*-PCR was associated with a lower CSF glucose, and a higher CSF cell count and protein, a longer duration of illness, and more frequent history of previous TB treatment (**table 2**). Clinical features of this group of patient were in accordance with that of TB meningitis. On presentation 44% had decreased level of consciousness, 28% had fever, 86% had neck stiffness, 32% had motor deficit, and 22% had cranial nerve palsies. 14% of these patients were HIV-positive. Among 21 HIV-infected patients with negative *M. tuberculosis* culture, similar differences were found, although none were significant.

**Table 2:** CSF and clinical characteristics of 166 HIV-negative patients with suspected TB-meningitis according to culture and PCR-result.

	Culture positive (n = 88)	Culture negative (n = 78)		
		PCR positive (n = 39)	PCR negative (n = 39)	p-value
Duration of illness (days) – median (IQR)	14 (7–30)	14 (7–21)	8 (6–14)	.104
History of TB – no. (%)	13/86 (15.1)	12/38 (31.6)	5/36 (13.9)	.068
**CSF**				
Cells/mL – median (IQR)	136 (47–262)	66.5 (7–121)	11 (0.8–334.8)	.302
PMN % – median (IQR)	37 (20–62)	13 (0.5–52)	10 (0–44)	.750
MN % – median (IQR)	63 (38–80)	72 (41–95.5)	72 (30–95)	.423
Protein (mg/dL) – median (IQR)	160 (63–370)	125 (40–342.5)	55 (40–412.5)	.620
Glucose (mg/dL) – median (IQR)	16 (9.8–32.0)	36.5 (18.8–48.8)	48 (37–60.8)	.026
CSF:blood glucose ratio – median (IQR)	0.13 (0.09–0.24)	0.34 (0.17–0.46)	0.46 (0.34–0.56)	.017

Three culture negative/PCR positive patients were not included because microscopy was positive. PMN = polymorphonuclear cell, MN = mononuclear cell, IQR = interquartile range.

## Discussion

In this study real time IS*6110*-targeted PCR had a high positivity rate (68%) among patients with suspected TB meningitis, higher than *M. tuberculosis* microscopy (11%), and *M. tuberculosis* culture using solid (36%) and liquid (44%) media, both among HIV-infected and non-infected subjects. CSF characteristics and clinical data of culture-negative/PCR-positive patients were in line with a diagnosis of TB meningitis, and specificity of the IS*6110*-PCR was 100% in a control group of patients with alternative diagnoses.

Untreated TB meningitis is almost uniformly lethal and delay in treatment is associated with increased mortality and morbidity. Unfortunately, CSF microscopy has poor sensitivity, while *M. tuberculosis* culture is slow and therefore has a limited role in decisions about treatment of possible TB meningitis. *M. tuberculosis* PCR has the potential to be the ideal tool for rapid diagnosis of TB meningitis. Unfortunately, commercial PCR tests from Roche, Abbott, and Genprobe before 2003 only have a moderate sensitivity for diagnosis of TB meningitis, as summarized in a systematic review [8]. Some studies have reported higher sensitivity for in-house PCR assays, but their role in diagnosis of TB meningitis remains uncertain. In the present study we evaluated in-house real time IS*6110*-PCR in CSF samples from a well characterised cohort of 230 meningitis patients, larger than previous series [18], [19]. The high prevalence of TB meningitis in our cohort was confirmed bacteriologically in the majority of patients, more than in most published case series [18], [20].

In-house IS*6110*-PCR had a higher positivity rate (68%) in our cohort compared to many other case series evaluating in-house PCR, some using IS*6110* as a target [6], [7], [21], [22], [23]. This may have been due to the use of large volume CSF samples (5–10 ml), as was nicely shown in an earlier study [5], and IS*6110*, which has multiple copies present in the genome of *M. tuberculosis* complex, as the PCR target [16]. We used PhHV as an inhibition control in each sample to rule out the possibility of PCR inhibition with a false negative PCR result. The positivity rate of IS*6110*-PCR in our study was lower among HIV-infected patients, in line with a study from India [24], and possibly due to the fact that cerebral toxoplasmosis may mimic TB meningitis in the absence of neruradiology, as we have recently shown [25], underlining the need for more extensive microbiological testing in HIV-infected patients. Compared with culture, IS*6110*-PCR had a sensitivity of 92%, in line with some earlier previous studies [3], [21], [26] but superior compared to some other studies which reported sensitivity rates of 32–75% [7], [12], [22], [23], [27], [28]. Two patients with cryptococcal meningitis, and one with bacterial meningitishad a positive IS*6110*-PCR result. Mixed infections of mycobacterial with cryptococcal meningitis [25], [29] and bacterial meningitis have been reported previously [24], [30]. The presence of *M. tuberculosis* DNA in our patient with bacterial meningitis was confirmed by *M. tuberculosis* spoligotyping.

The detection of IS*6110* PCR in culture-negative patients (n = 46) was often (40%) supported by TB outside the CNS or a good response to TB treatment. In addition, some patients had been taking TB drugs, which is known to sterilize CSF cultures within days [31]. The higher positivity of IS*6110*-PCR compared to culture (although not statistically significant compared with liquid culture) in these patients may have been due to amplification of DNA from non-viable organisms. Further support for the specificity of IS*6110*-PCR was derived from a closer examination of the group of culture-negative patients. Those with positive PCR results usually had a lower CSF glucose, raised protein level, and more pronounced pleiocytosis. A previous history of TB was more common in patients with a positive IS*6110*-PCR result (33% vs 14%), and duration of illness was typically longer (median 14 days vs 7 days). Obviously, the sensitivity of this assay will be lower in areas with a substantial prevalence of *M. tuberculosis* strains with no or only a few IS*6110* copies. However, this seems to be relatively rare, and multiple copies of IS*6110* were present in all strains from a previous study we conducted in Indonesia [32].

The positive IS*6110*-PCR results obtained from those patients could not be due to amplicon contamination as physical separation rooms were used for sample processing, PCR setup, amplification process, carefull handling procedure, the use of barrier tips, dUTP-uracil glycosylase system, and unambiguous negative results from our negative IS*6110*-PCR controls. Real time PCR allows direct observation of amplicon reaction without the need to open PCR tubes, thereby avoiding the possibility of amplicon contamination. Finally, none of the patients in the control group with non-infectious CNS disease had a positive IS*6110*-PCR result.

The strength of our study is the validation of in-house IS*6110*-PCR in a large group of TB meningitis patients, much larger than most previously published series. Other strenghts include the use of comparative *M. tuberculosis* culture (solid and liquid) and microscopy, a work-up for other pathogens, and the fact that laboratory results were linked with clinical data. Unfortunately no direct comparison could be made with a commercial PCR. Despite this limitation, we can conclude that in-house PCR targetting IS*6110* is a very sensitive and specific method for diagnosis of TB meningitis, which may speed up diagnosis and timely treatment of this deadly disease. It should be noted that this assay, evaluated in a single highly-qualified laboratory, may not perform equally well in other settings.
